# Changes of Corneal Wavefront Aberrations in Dry Eye Patients after Treatment with Artificial Lubricant Drops

**DOI:** 10.1155/2016/1342056

**Published:** 2016-03-14

**Authors:** Ning Lu, Fangyu Lin, Zhu Huang, Qin He, Wei Han

**Affiliations:** Department of Ophthalmology, First Affiliated Hospital, College of Medicine, Zhejiang University, Hangzhou 310003, China

## Abstract

*Purpose.* To evaluate the corneal aberration changes in dry eye patients after treatment with artificial eye drops.* Methods.* Thirty mild to moderate dry eye patients treated with artificial eye drops and twenty comparable dry eye patients were recruited as controls. Anterior corneal aberrations over 3 mm and 5 mm analytical zones including total, 3rd to 5th high order aberrations (HOAs), spherical aberration (SA), and vertical (V-coma) and horizontal coma (H-coma) obtained from corneal topography data at baseline and 2 weeks after treatment were evaluated.* Results.* For 3 mm zone, trefoils, V-coma, H-coma terms, and 3rd and 5th HOAs were significantly decreased (*p* < 0.05) in the treatment group. For 5 mm zone, instillation of eye drops reduced H-coma, SA terms, 3rd to 5th orders, and total HOAs all showed a significant decrease (*p* < 0.05). The root mean square analysis of the Zernike terms also showed similar statistical results. For control group, all individual terms and total HOAs did not have significant changes over 3 mm and 5 mm zones (*p* > 0.05).* Conclusions.* Treatment with artificial eye drops can effectively improve the corneal optical quality of dry eye patients by ameliorating the HOAs of anterior corneal surface.

## 1. Introduction

Dry eye is one of the most common diseases in ophthalmology clinic with symptoms of ocular discomfort such as eyestrain, ocular dryness, redness, foreign body sensation and pain, and poor night vision, resulting from tear deficiency or abnormal tear film function [1]. With computer technology having penetrated each corner of our society, more and more people are suffering from the disorder of dry eye throughout the world. The precorneal tear film is a critically important factor contributing to stability of the ocular optical quality [2]. When tear film, the most anterior refractive surface of the eye, breaks up, the optical surface becomes irregular and may introduce additional aberrations into the optical system [3, 4]. Several previous studies support this hypothesis that increased irregularity and dynamic changes of tear film caused by dry eye showed greater optical aberrations compared with normal eyes [5–8], and these changes might cause deterioration of visual function and optical quality [4, 9].

An intact and regular tear film is essential for high quality retinal images and the deteriorated tear film can cause degraded visual function. Artificial tears eye drops are used to lubricate the ocular surface and relieve the symptoms in dry eye patients and thus are expected to improve the optical quality by making a smoother corneal surface. Most patients report that their visual function and photophobia symptoms were improved [10] after instillation of artificial tears eye drops. Previous researches have reported that there was significant improvement of optical quality as aberrations associated with irregular tear film were decreased significantly after artificial tears instillation [1, 3, 11]. Accordingly, wavefront aberrometry is an essential and objective approach to detect the deterioration of optical quality [12]. Several studies using corneal topography [2, 13, 14] and Hartmann-Shack wavefront aberrometer [15–17] shed valuable light on the optical quality changes for dry eye cases.

To further investigate the specific corneal aberration changes and their potential relation with the clinical symptoms of dry eye, this study was designed to analyze the corneal high order aberrations (HOAs) and the visual quality using approach of corneal topography in a group of dry eye patients before and after treatment with artificial tear drop.

## 2. Patients and Methods

### 2.1. Participants

This prospective, randomized study was conducted in the First Affiliated Hospital of Zhejiang University, Hangzhou, China. The research adhered to the tenets of the Declaration of Helsinki. Written informed consent was received from all patients. A total of 52 patients diagnosed with mild to moderate dry eye disease were involved in this study, according to the criteria of the Dry Eye Workshop 2007 [18].

Basically, only mild to moderate dry eye patients were recruited in this study. The inclusion criteria were as follows: young adult patients 20–40 years old, who complained about dry eye symptoms for at least 1 month. One or multiple symptoms were involved: ocular dryness, burn and foreign body sensation, visual blur, or discomfort like night vision, reading, and so forth. All eyes had a best spectacle-corrected visual acuity (BCVA) of 20/30 or better. Tear film breakup time (TBUT) was ≤10 s for each eye, and Schirmer I test (without anesthesia) was ≥10 mm wetting/5 minutes for each eye, with mild (<10 spots) or without positive results on corneal fluorescein (FL) staining. For both treatment and control groups, only right eye was used for the measurements. The spherical equivalence (SE) for treatment subjects was −1.59 ± 1.16 D (−6.25– + 0.50), while the SE for control subjects was −1.31 ± 1.56 D (−5.75– + 1.00). SE was calculated as sphere diopters plus half cylinder. SE of the right eyes for each subject was used for analysis (Table 1).

Exclusion criteria were as follows: unilateral dry eye patients, contact lens wearer, permanent occlusion of lacrimal puncta in either eye, significant meibomian gland disease (MGD), blepharitis, severe dry eye diseases with Steven-Johnson or Sjögren's syndrome, pterygium and other coexisting ocular diseases, a history of ocular surgery, and so forth. Patients with any previous using of eye drops for dry eye or other ocular disorders, any other systematic diseases like diabetes, thyroid disease, and rheumatoid disease were excluded.

### 2.2. Experimental Protocol

Pretreatment and posttreatment examination including BCVA (LogMar), slit-lamp examination, TBUT, FL staining, and Schirmer I test were performed. Air-tear film interface characterizations were obtained from corneal topography with a CSO topography system (C.S.O. SRL Modi, Italy). To prevent diurnal changes, all examinations were performed between 9 and 11 a.m. The chief subjective complaints of eye dryness, foreign body sensation, and vision disturbance (such as blur, discomfort, and night vision) were also recorded.

For each subject, TBUT and staining with FL were performed. A FL strip was used to touch the inferior fornix when patient looked up. The patient was instructed to blink several times to ensure adequate mixing of the dye and then hold the eye open. A cobalt blue illumination of the slit-lamp was used, and the interval in seconds between the last complete blink and the appearance of the first random corneal black spot was recorded as tear film breakup time. The test was repeated three times, using a chronometer, and the mean value of the measurements was calculated.

The patients were told to blink several times before corneal topographic images were captured. To rule out the confounding from tear film desiccation for the dry eye, the corneal images were captured just after blinking as quickly as possible (within 2-3 seconds). For each eye, the corneal topography measurement was performed three times consecutively and the average value was taken for analysis. Corneal topography showed a change in the number of contour lines, reflecting a variation in the aberration of the anterior surface of tear film. The topography data were transformed to Zernike wavefront aberration polynomials based on the Holladay formula. Corneal monochromatic high order aberrations (HOAs) in the central 3 and 5 mm zones from 3rd up to 5th order were used for analysis used by expanding the set of Zernike polynomials in this study. For specific terms of trefoil, vertical/horizontal coma and spherical aberration (SA), the Zernike coefficients were used for analysis. Root mean square (RMS) was calculated to analyze the total 3rd, 4th, and 5th wavefront aberrations, respectively.

Schirmer I test was performed last so that the ocular irritation caused by the strip would not interfere with other results. A Whatman 41 paper strip was placed in the lateral and middle thirds of lower conjunctiva sac away from the cornea without topical anesthesia. The measure was recorded after 5 minutes with the eyes closed (at least 30 minutes after TBUT and staining scoring). The amount of wetting of the strip was recorded in millimeters. All examinations were made in the environment with stable humidity and temperature.

The fifty-two subjects were then reassigned randomly into two groups, the treatment group and the control group. Treatment group underwent instillation of 0.1% sodium hyaluronate eye drops (HYCOSAN, EUSAN GmbH Co., Germany) four times a day. After 2 weeks, all the patients were requested to come back to hospital for examination. The patients were also questioned about each subjective dry eye symptom and any ocular side effects related to the eye drops.

### 2.3. Statistical Analysis

Zernike polynomials transformed from corneal data were used to determine the 3rd, 4th, and 5th aberrations, SA (*Z*4,0), trefoil (*Z*3, −3)  (*Z*3,3), and coma (*Z*3, −1)  (*Z*3,1) aberrations of anterior corneal surface through the build-in Holladay formula. The point-spread function (PSF) is a basic parameter to evaluate image quality, which is defined as the light distribution function of a point light source through an optical system [14]. The PSF and simulated wavefront aberration pattern were also used to evaluate the visual quality of the dry eye patients.

Statistical analysis was performed with SPSS software version 19.0 (SPSS, Inc., Chicago, IL). Independent *t*-tests were used to compare data between the two groups, and paired *t*-tests were used for statistical analysis to compare data before and after each treatment within groups. The Zernike coefficients and the relevant absolute values (root mean square, RMS) were both analyzed. All values are given as the mean ± standard deviation. A *p* value less than 0.05 was considered statistically significant.

## 3. Results

This study evaluated 52 right eyes of 52 patients. The treatment group included 30 eyes (14 men and 16 women) with the mean age 31.03 ± 4.73 years, ranging from 23 to 39 years. Control group included 22 eyes (12 men and 10 women) with the mean age 29.00 ± 5.30 years, ranging from 21 to 38 years (*t* = 1.455, *p* = 0.152) (Table 1).

At baseline, no significant differences were detected between the treatment and control groups for all objective and subjective dry eye-related examinations, including BCVA, TBUT, Schirmer I score, FL stain positive, eye dryness, and visual discomfort. However, after two weeks of treatment, there was significant amelioration of the symptoms for the treatment group when compared with controls, except BCVA and Schirmer I test (*p*′ < 0.01) (Table 1). BCVA LogMar (0.10 ± 0.05) were significantly better than the baseline (0.14 ± 0.09) after 2 weeks (*t* = 4.067, *p* < 0.001). Most of the subjects had refractive error with mild to moderate myopia, which might be responsible for the mean BCVA slightly less than 20/25. However, the factor of dry eye also should be taken into account according to the data of significantly improved BCVA in the treatment group (Table 1). The TBUT after treatment was 6.27 ± 1.20 s, significantly longer than the baseline level (4.93 ± 1.31 s, *t* = −7.345, and *p* < 0.001) and that in the control group (5.18 ± 1.05 s, *t* = 3.386, and *p* < 0.001). Schirmer I test did not show significant improvement. Compared with the baseline, the corneal punctuate FL positive staining rate also significantly decreased after 2 weeks of treatment, from 60% to 16.7% (Table 1). Meanwhile, for the control group, all examinations did not have significant differences after 2 weeks (*p* > 0.05) (Table 1).

**Figure 1:**
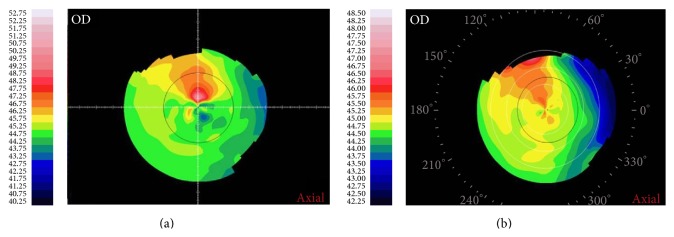
Corneal topography image of one patient receiving treatment (axial power map).

Figure 1 shows an example of corneal topography map before (a) and after (b) 2-week artificial tear instillation in one patient. The corneal maps are centered at the pupil center. The patient reported a significant vertical diplopia symptom before treatment, which was ameliorated after treatment.

**Figure 2:**
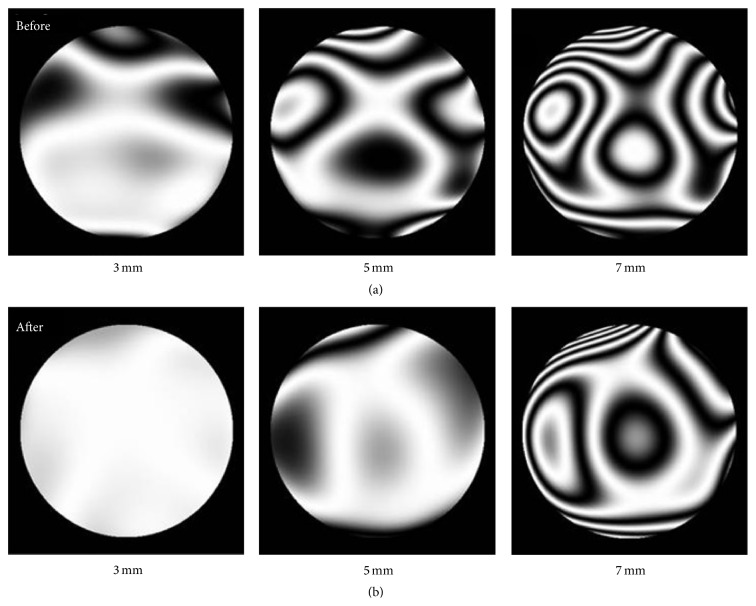
Corneal wavefront aberration contour plots of one patient receiving treatment. The patterns of (a) denote the results before treatment, while those of (b) denote the results after treatment.

**Figure 3:**
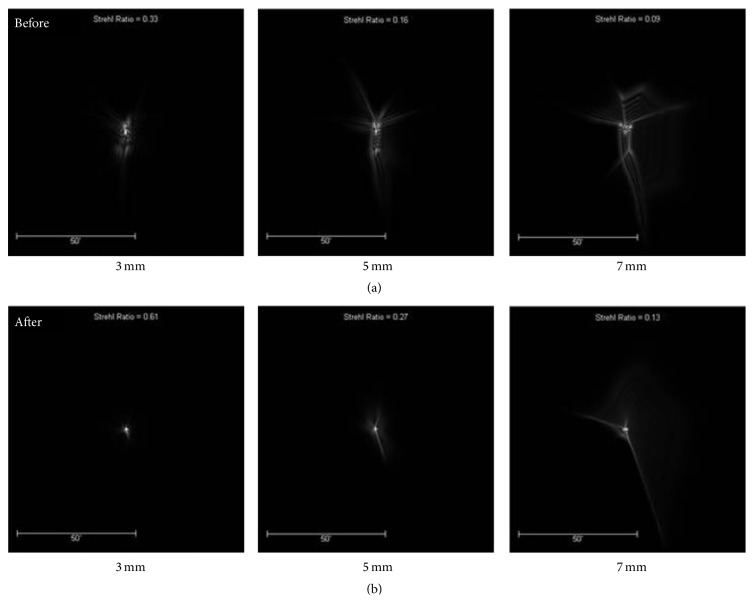
The PSF image of one patient receiving treatment with artificial tear drops. The patterns of (a) denote the results before treatment, while those of (b) denote the results after treatment.

Figure 2 shows an example of corneal wavefront aberration contour plots over 3 mm, 5 mm, and 7 mm pupil zone before and after treatment, respectively. The HOAs from 3rd to 5th were involved in the analysis plots. Compared with pretreatment plots, the number of contour lines for posttreatment becomes apparently less over all diameter zones, indicating a better quality of tear film.  Figure 3 shows the relevant PSFs results with the best correction of any defocus and astigmatism. PSFs represent a function for describing the optical performance.

All PSFs are more concentrated and have better contrast after two weeks than that at baseline and the minimum PSF was observed at 3 mm diameter zone (Figure 3).

For 3 mm analytical zone, all Zernike terms of 3rd order and three Zernike terms (*Z*(4, −2), *Z*(4,2), and *Z*(4,4)) of 4th order showed significant changes after two weeks of treatment (*p* < 0.05). For 5 mm zone, only *Z*(3, −1), *Z*(3,1), *Z*(3,3), and *Z*(4,0) terms showed significant difference at baseline and after treatment (*p* < 0.05) (Table 2). No significant changes of aberration terms were found for the control group (*p* > 0.05) (Table 2).

The RMS values analysis showed significant changes in *Z*(3, −3), *Z*(3,1), *Z*(3,3), and *Z*(4, −2) terms for 3 mm zone (*p* < 0.05). For 5 mm zone analysis, the RMS of *Z*(3, −3), *Z*(4, −4), *Z*(4, −2), and *Z*(4, 0) terms showed significant changes (*p* < 0.05). Similarly, no significant changes of RMS for all Zernike terms were found for the control group (*p* > 0.05) (Table 3).

**Figure 4:**
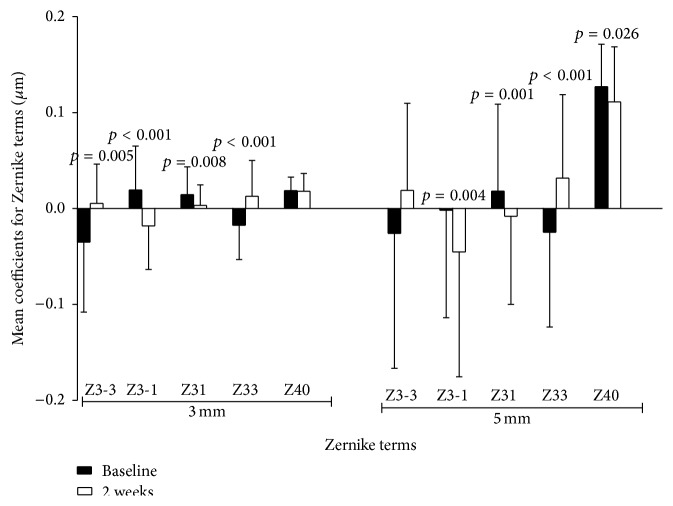
Zernike terms in treatment group. Vertical coma and horizontal coma decreased significantly at a 3 mm zone. Vertical coma, horizontal coma, and SA had significant mathematical decrease at 5 mm zone.

Figure 4 shows the intuitive comparison results of Zernike coefficients for vertical coma, horizontal coma, and SA terms over 3 mm and 5 mm zones between baseline and 2 weeks for the treatment group. For 3 mm zone analysis, the vertical coma and horizontal coma terms at 2 weeks after treatment (−0.0181 ± 0.0454 *μ*m and 0.0032 ± 0.0215 *μ*m) were significantly decreased (*p* < 0.001 and *p* = 0.008), compared with those at baseline (0.0192 ± 0.0458 *μ*m and 0.0145 ± 0.0289 *μ*m, resp.). The SA term did not show any significant differences (*t* = 0.1470, *p* = 0.884). For 5 mm zone, vertical coma, horizontal coma, and SA at 2 weeks all had mathematical and statistically significant decrease (−0.0453 ± 0.1301 *μ*m, −0.0081 ± 0.0919 *μ*m, and 0.1111 ± 0.0575 *μ*m; *p* = 0.004, *p* = 0.001, and *p* = 0.026, resp.), compared with those at baseline (−0.0018 ± 0.1121 *μ*m, 0.0181 ± 0.0907 *μ*m, and 0.127 ± 0.0442 *μ*m).

**Figure 5:**
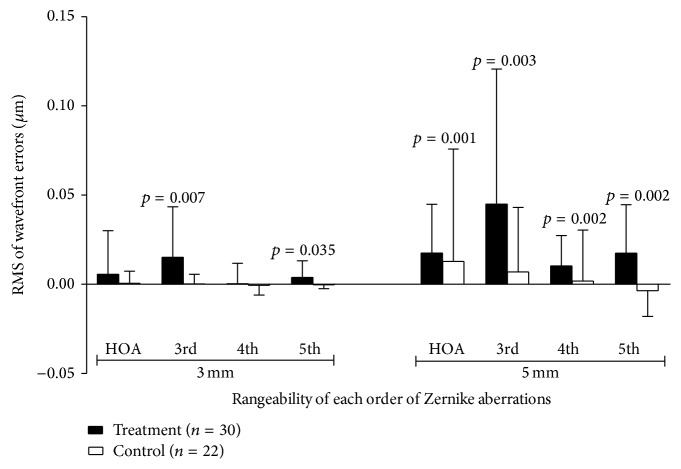
Rangeability of HOAs (baseline minus 2 weeks) for the treatment and control groups. A positive value of RMS rangeability indicates a decreasing of HOAs amplitude after treatment, and vice versa. For 3 mm zone, the 3rd and 5th HOAs decreased significantly after two weeks of artificial tears eye drops treatment. For 5 mm zone, the 3rd, 4th, 5th, and total HOAs all showed a significant decrease after 2 weeks of treatment.

Figure 5 showed the rangeability plot of HOAs (baseline minus two weeks' results) in treatment and control groups. Column above zero in *y*-axis denotes the RMS decreasing of HOAs two weeks after treatment, otherwise denoting increasing of RMS. For the treatment group, the RMS of 3rd and 5th HOAs (rangeability was 0.0151 ± 0.0283 *μ*m, 0.0038 ± 0.0094 *μ*m, resp.) was decreased significantly after treatment (*p* = 0.007, *p* = 0.035) over 3 mm zone, while the RMS of 3rd, 4th, 5th, and total HOAs over 5 mm zone (rangeability was 0.0449 ± 0.0758 *μ*m, 0.0103 ± 0.0170 *μ*m, 0.0174 ± 0.0273 *μ*m, and 0.0175 ± 0.0273) all showed a significant decrease 2 weeks after treatment (*p* = 0.003, 0.002, 0.002, and 0.001, resp.).

For the control group, all the Zernike terms of 3rd and 4th HOAs as well as the 3rd, 4th, 5th, and total HOA over 3 mm and 5 mm zones did not show significant differences between baseline and 2 weeks after treatment (*p* > 0.05) (Figure 5) (Table 2).

## 4. Discussion

Many people with dry eye symptoms complain of blurry vision and related visual symptoms even with normal visual acuity, which are most possibly caused by large HOAs. Compared with low order aberrations, HOAs may contribute to a lower proportion of total optical aberrations, but they play a vital role in visual quality and can not be corrected by spectacle [19]. As a crucial medium in refractive system, cornea anterior surface produces almost 80% portion of whole ocular refraction power. Corneal aberrations mainly derive from anterior cornea surface, whereas posterior cornea surface creates relatively stable aberration which contributes little to corneal aberrations. In terms of anterior cornea surface, tear film is the most anterior covering layer forming interface with the air. Thus one can expect that the deteriorated tear film caused by dry eye may result in an irregular anterior cornea surface and considerably degrade the visual quality. Previous researches reported that dry eye patients had almost 2.5 times larger corneal aberrations than normal people because of irregularities across the corneal surface due to the change of tear film [5, 20]. This anterior surface irregularity was also found to lead to a time-dependent dynamic change in retinal image quality [7]. The present work focused on the influence of treatment with artificial eye drops in the anterior corneal aberrations and relevant visual function for dry eyes.

Corneal wavefront aberrations were found to have wide variation among individuals [21] and such substantial individual variation can cause the dynamic change of the Zernike aberrations [22]. This was also demonstrated in our dry eye data. Some aberration terms were still highly variable with large standard deviation and wide range (Figures 4 and 5). Besides the factor of tear film fluctuation, we thought this might also be ascribed to the different lifestyle and career environment of the subjects recruited. Nevertheless, effort had been made in this study to obtain relatively consistent ocular surface data as far as possible, including subjects matching, careful and skillful data image acquiring, average value taken from three measures, and constant room conditions.

The 3rd- and 4th-order aberrations were thought to play important role in the central visual quality. The composition of corneal aberration is also essentially consistent with entire ocular optic system, in which SA have more considerable effect on visual quality, especially night vision, than coma and trefoil. For coma terms, horizontal coma was thought to have a more negative influence than vertical coma [23].

SA results from different focusing capability between central and peripheral cornea and is mostly positive [21, 24–26]. Several studies [2, 20, 22] have reported the dynamic change of SA in a short time period after blinking and found significant increase about 10 seconds after blink, suggesting the desiccated tear film layer can influence the corneal optical quality. The average SA in our study group at baseline had positive value and was relatively smaller compared with those found in other studies [27, 28], which might be due to variation of individuals and measurements among different studies. Such change in SA could be a consequence of a general tendency for the tear film tending to thin more rapidly at the center than peripheral cornea zone due to the tear film evaporation [29]. This process may change corneal surface shape from prolate to oblate and thus introduce more positive SA [7, 22]. In this study, only SA of 5 mm zone demonstrated mild significant decreasing from the baseline level to 2 weeks after treatment (Table 2, Figure 4), indicating that eye drops might extend the tear film holding time and reduced the anterior surface irregularities over a larger cornea zone rather than 3 mm central zone. This could also be related to the ameliorated visual symptoms such as night vision/reading disturbance as reported by the patients (Table 1). Longer treatment with eye drop might expose more significant changes. Nevertheless, our data of two-week posttreatment were comparable to the previous studies. Montés-Micó et al. [14] found reduction by a factor of approximately 2.5 in SA, coma aberrations, and total HOAs immediately after artificial tear instillation and the decrease was maintained in 10 minutes. Koh et al. [30] and Kaido et al. [31] both reported that SA was significantly lowered after diquafosol tetrasodium administration at 1 month.

Asymmetric spreading of tear film on corneal surface may result in coma-like aberrations [29]. Several investigations have shown the large variations of coma terms in both absolute value and direction in normal eyes. Piñero et al. [32] reported 0.001 ± 0.225 vertical and −0.001 ± 0.128 horizontal coma for anterior corneal surface in 15 eyes using Pentacam; in Wang et al.'s [21] research including 228 eyes, the data was −0.083 ± 0.183 and 0.000 ± 0.193, respectively, by CTView program. In a Chinese adult population, Li et al. [33] reported −0.043 ± 0.161 and 0.070 ± 0.115 for ocular vertical and horizontal coma using iTrace Dynamic Laser Refraction system. In this work, the Zernike coefficients of vertical and horizontal coma over 3 mm and 5 mm zones were all decreased significantly to a negative direction after 2 weeks (Table 2, Figure 4). The coma aberrations changes might be caused by the gravitational effects or lid pressure and the quality of tear film, leading to differential thinning of tear film across corneal area vertically or horizontally [5, 7]. However, it is uncertain what the underlying mechanism responsible for such negative shift of the coma coefficients is. But one still can image the fact that it is relevant to the improved visual quality (Table 1) after treatment at this stage.

Similarly, the other two important 3rd-order terms, trefoil of *Z*(3, −3) and *Z*(3,3), also showed significant changes after treatment, particularly for 3 mm zone. The changes of trefoil aberration might also be important to the optical quality improvement and hence the better visual function outcome (Figures 2 and 3).

Total HOAs are index for evaluating general optical quality. HOAs were found to increase in a short time period after artificial eye drop administration, which might be ascribed to the corneal regularity immediately after eye drops instillation [1, 34, 35]. But after long term treatment with artificial tear drop, the HOAs were significantly ameliorated, denoting the importance of the tear film for ocular HOAs [29–31, 34]. In this study, we found a significant decrease of RMS for total and 3rd, 4th, and 5th HOAs over 5 mm cornea zone two weeks after treatment; meanwhile significant decrease of RMS was found for 3rd- and 5th-order aberrations over 3 mm cornea zone (Figure 5). These findings indicated the major HOAs on the central corneal surface were considerably ameliorated after two weeks of treatment with artificial tear drops, particularly over a larger zone of 5 mm. The markedly less divergence of PSF and wavefront aberration image provided an intuitive evidence of therapeutic effect on corneal optical quality for the dry eye patients after treatment with eye drop administration (Figures 2 and 3). Denoyer et al. reported significant degraded corneal and ocular HOAs, particularly 3rd aberrations, 10 seconds after blinking in dry eye patients [36]. For our treatment group, the eye drops instillation improved the tear film quality in terms of intact, regularity, and breaking up, as demonstrated by the relevant clinical test results (Table 1). Typically, the TBUT index increased significantly to longer than 6 s at two weeks, indicating a better tear film stability after treatment (Table 1). This should contribute to the improvement of corneal aberrations and hence be causative for the coupled relief of subjective visual symptoms as reported by our patients, like blur, reading, or night vision discomfort (Table 1 and Figure 3). Therefore, the data in this work suggested a logical association between effect of artificial tear drop treatment and visual symptoms improvement for dry eye individuals.

For RMS analysis over 3 and 5 mm zone, the changes between pre- and posttreatment were almost the same as the data of the coefficients analysis (Tables 2 and 3). This could be ascribed to the paired *t*-test algorithm used here, which should be robust for the individual self-change comparison. The RMS of *Z*(4,0) term over 5 mm zone gave the same statistical results as coefficient analysis, because all *Z*(4,0) values were positive. It is notable that almost all RMS values of each Zernike term were larger than their relevant Zernike coefficients (Table 3), denoting the avoidance of cancellation among the coefficients due to the value azimuth and hence the better description for the aberrations amplitude.

Limitations exist in our study. We only enrolled young adult subjects with mild to moderate dry eye, but dry eye disorder is common in all age bands and includes more severe grades. In addition, we did not perform stratification analysis based on the subjective measurements reported by patients, since almost all patients (more than 90%) reported one or more improved subjective symptoms after two weeks of treatment and hence subgrouping of the patients was not viable for statistical analysis. Further studies with larger sample size, more type/grade of dry eye, and longer follow-up time are desirable to reveal more specific picture. In the present study, we evaluated the individual HOA terms and total HOAs changes from 3rd to 5th orders. Our data confirmed the better central corneal HOAs, particularly for some important HOA terms like SA and coma, coupled with the ameliorated dry eye symptoms and visual functions after lubricant eye drop instillation. Our finding also suggested the potential importance of corneal aberrometry in dry eye assessment such as diagnosis and treatment effect evaluation.

## Figures and Tables

**Table 1:** Clinical data analysis for the dry eye patients at baseline and two weeks after treatment.

	Treatment group (*n* = 30)			Control group (*n* = 22)			*p*′
	Baseline	Two weeks	*p*	Baseline	Two weeks	*p*	
Age (year)	31.03 ± 4.73 (23–39)		—	29.00 ± 5.30 (21–38)		—	>0.05
Male/female	14/16		—	12/10		—	>0.05
BCVA (LogMAR)	0.14 ± 0.09	0.10 ± 0.05	0.03	0.12 ± 0.09	0.11 ± 0.07	>0.05	>0.05
SE (diopter)^#^	−1.59 ± 1.16 (−6.25–+ 0.50)		—	−1.31 ± 1.56 (−5.75–+1.00)		—	>0.05
TBUT (s)	4.93 ± 1.31	6.27 ± 1.20	<0.01	4.95 ± 1.13	5.18 ± 1.05	>0.05	<0.01
Schirmer I score (mm)	11.80 ± 1.77	12.10 ± 1.42	>0.05	11.14 ± 1.73	11.36 ± 1.56	>0.05	>0.05
Fluorescein stain positive (eye)^§^	18 (60%)	5 (16.7%)	<0.01	12 (54.55%)	13 (57%)	>0.05	<0.01
Eye dryness /burn/foreign body sensation (eye)^§^	25 (83.3%)	11 (36.7%)	<0.01	18 (81.8%)	19 (86.4%)	>0.05	<0.01
Vision disturbance (blur/discomfort/night vision) (eye)^§^	21 (70%)	9 (30%)	<0.01	15 (68.2%)	16 (72.8%)	>0.05	<0.01

^**#**^SE denotes spherical equivalence, which was calculated as sphere diopters plus half-cylinder diopters. SE of the right eyes for each subject was used for analysis.

^§^The numbers for these tests denote the number of eyes showing positive results and the percentage in parenthesis denotes the rate of positive results.

*p* indicates the significant level of *t*-test within treatment or control groups between baseline and two weeks after treatment.

*p*′ indicates the significant level of *t*-test between treatment and control groups after two weeks.

**Table 2:** Comparison of 3 mm and 5 mm zones Zernike terms of 3rd and 4th HOAs between baseline and 2 weeks after treatment.

	3 mm zone				5 mm zone			
	Treatment group (*n* = 30)		Control group (*n* = 22)		Treatment group (*n* = 30)		Control group (*n* = 22)	
	Baseline	2 weeks	Baseline	2 weeks	Baseline	2 weeks	Baseline	2 weeks
*Z*_3_^−3^	−0.0351 ± 0.0729^*∗∗*^	0.0052 ± 0.0411^*∗∗*^	−0.0044 ± 0.0154	0.0030 ± 0.0209	−0.0259 ± 0.1407	0.0189 ± 0.0908	−0.0159 ± 0.0595	−0.0038 ± 0.0613
*Z*_3_^−1^	0.0192 ± 0.0458^*∗∗*^	−0.0181 ± 0.0454^*∗∗*^	−0.0101 ± 0.0353	−0.0095 ± 0.0293	−0.0018 ± 0.1121^*∗∗*^	−0.0453 ± 0.1301^*∗∗*^	−0.0321 ± 0.1487	−0.0214 ± 0.1165
*Z*_3_^1^	0.0145 ± 0.0289^*∗∗*^	0.0032 ± 0.0215^*∗∗*^	−0.0099 ± 0.0187	−0.0069 ± 0.0200	0.0181 ± 0.0907^*∗∗*^	−0.0081 ± 0.0919^*∗∗*^	−0.0272 ± 0.0805	−0.0228 ± 0.0809
*Z*_3_^3^	−0.0176 ± 0.0356^*∗∗*^	0.0128 ± 0.0373^*∗∗*^	−0.0022 ± 0.0094	0.0018 ± 0.0132	−0.0249 ± 0.0987^*∗∗*^	0.0316 ± 0.0872^*∗∗*^	−0.0190 ± 0.0384	−0.0076 ± 0.0534
*Z*_4_^−4^	−0.0025 ± 0.0176	−0.0023 ± 0.0160	0.0034 ± 0.0086	0.0015 ± 0.0087	0.0014 ± 0.0607	−0.0117 ± 0.0264	0.0176 ± 0.0363	0.0142 ± 0.0422
Z_4_^−2^	−0.0038 ± 0.0051^*∗∗*^	0.0054 ± 0.0074^*∗∗*^	0.0002 ± 0.0051	0.0005 ± 0.0069	0.0020 ± 0.0237	−0.0018 ± 0.0122	−0.0064 ± 0.0227	−0.0061 ± 0.0311
*Z*_4_^0^	0.0186 ± 0.0142	0.0180 ± 0.0184	0.0126 ± 0.0069	0.0089 ± 0.0121	0.1270 ± 0.0442^*∗*^	0.1111 ± 0.0575^*∗*^	0.1356 ± 0.0499	0.1231 ± 0.0469
*Z*_4_^2^	0.0042 ± 0.0171^*∗*^	−0.0072 ± 0.0217^*∗*^	−0.0044 ± 0.0107	−0.0036 ± 0.0103	0.0031 ± 0.0337	−0.0013 ± 0.0295	−0.0151 ± 0.0376	−0.0183 ± 0.0457
*Z*_4_^4^	−0.0066 ± 0.0200^*∗*^	0.0034 ± 0.0242^*∗*^	−0.0002 ± 0.0120	0.0009 ± 0.0124	−0.0080 ± 0.0611	−0.0077 ± 0.0624	−0.0105 ± 0.0385	0.0009 ± 0.0519

^*∗*^ *p* < 0.05, ^*∗∗*^ *p* < 0.01 between baseline and two weeks after treatment.

**Table 3:** Comparison of 3 mm and 5 mm zones RMS of Zernike terms of 3rd and 4th HOAs between baseline and 2 weeks after treatment.

	3 mm zone				5 mm zone			
	Treatment group (*n* = 30)		Control group (*n* = 22)		Treatment group (*n* = 30)		Control group (*n* = 22)	
	Baseline	2 weeks	Baseline	2 weeks	Baseline	2 weeks	Baseline	2 weeks
*Z*_3_^−3^	0.0479 ± 0.0349^*∗∗*^	0.0280 ± 0.0301^*∗∗*^	0.0213 ± 0.0232	0.0233 ± 0.0300	0.1135 ± 0.0846^*∗*^	0.0703 ± 0.0566^*∗*^	0.0296 ± 0.0367	0.0308 ± 0.0553
*Z*_3_^−1^	0.0342 ± 0.0355	0.0353 ± 0.0333	0.0271 ± 0.0533	−0.0295 ± 0.0611	0.0918 ± 0.0620	0.1099 ± 0.0809	0.0401 ± 0.0882	0.0351 ± 0.0912
*Z*_3_^1^	0.0239 ± 0.0214^*∗*^	0.0150 ± 0.0155^*∗*^	0.0175 ± 0.0198	−0.0159 ± 0.0261	0.0743 ± 0.0534	0.0669 ± 0.0623	0.0588 ± 0.0952	0.0600 ± 0.0799
*Z*_3_^3^	0.0286 ± 0.0272^*∗∗*^	0.0399 ± 0.0121^*∗∗*^	0.0222 ± 0.0154	0.0199 ± 0.0232	0.0765 ± 0.0657	0.0786 ± 0.0473	0.0377 ± 0.0442	0.0426 ± 0.0912
*Z*_4_^−4^	0.0133 ± 0.0114	0.0115 ± 0.01116	0.0194 ± 0.0118	0.0155 ± 0.0287	0.0496 ± 0.0338^*∗∗*^	0.0227 ± 0.0175^*∗∗*^	0.0386 ± 0.0533	0.0440 ± 0.0482
*Z*_4_^−2^	0.0050 ± 0.0032^*∗*^	0.0070 ± 0.0028^*∗*^	0.0090 ± 0.0066	0.0083 ± 0.0092	0.0166 ± 0.0167^*∗∗*^	0.0090 ± 0.0083^*∗∗*^	0.0201 ± 0.0337	0.0241 ± 0.0551
*Z*_4_^0^	0.0198 ± 0.0124	0.0180 ± 0.0184	0.0168 ± 0.0175	0.0197 ± 0.0221	0.1270 ± 0.0441^*∗*^	0.1111 ± 0.0575^*∗*^	0.1356 ± 0.0499	0.1231 ± 0.0469
*Z*_4_^2^	0.0136 ± 0.0108	0.0146 ± 0.0174	0.0115 ± 0.0093	0.0126 ± 0.0103	0.0229 ± 0.0245	0.0235 ± 0.0173	0.0281 ± 0.0456	0.0253 ± 0.0537
*Z*_4_^4^	0.0176 ± 0.01115	0.0188 ± 0.0153	0.0059 ± 0.0092	0.0069 ± 0.0167	0.0498 ± 0.0351	0.0483 ± 0.0393	0.0405 ± 0.0523	0.0389 ± 0.0605

^*∗*^ *p* < 0.05, ^*∗∗*^ *p* < 0.01 between baseline and two weeks after treatment.
